# Evaluation of Radio-Protective Effect of Melatonin on Whole Body Irradiation Induced Liver Tissue Damage

**Published:** 2013-02-20

**Authors:** Alireza Shirazi, Ehsan Mihandoost, Ghazale Ghobadi, Mehran Mohseni, Mahmoud Ghazi-khansari

**Affiliations:** 1. Department of Medical Physics and Biomedical Engineering, Faculty of Medicine, Tehran University of Medical Sciences, Tehran, Iran; 2. Department of Medical Radiation Engineering, Science and Research Branch, Islamic Azad University, Tehran, Iran; 3. Department of Radiation Oncology, University Medical Center Groningen, University of Groningen, Groningen, The Netherlands; 4. Department of Radiology and Medical Physics, Faculty of Paramedicine, Kashan University of Medical Sciences, Kashan, Iran; 5. Department of Pharmacology, Faculty of Medicine, Tehran University of Medical Sciences, Tehran, Iran

**Keywords:** Radiation, Lipid peroxidation, MDA, GSH

## Abstract

**Objective:**

Ionizing radiation interacts with biological systems to induce excessive fluxes of free radicals that attack various cellular components. Melatonin has been shown to be a direct free radical scavenger and indirect antioxidant via its stimulatory actions on the antioxidant system.The aim of this study was to evaluate the antioxidant role of melatonin against radiation-induced oxidative injury to the rat liver after whole body irradiation.

**Materials and Methods:**

In this experimental study,thirty-two rats were divided into four groups. Group 1 was the control group, group 2 only received melatonin (30 mg/kg on the first day and 30 mg/kg on the following days), group 3 only received whole body gamma irradiation of 10 Gy, and group 4 received 30 mg/kg melatonin 30 minutes prior to radiation plus whole body irradiation of 10 Gy plus 30 mg/kg melatonin daily through intraperitoneal (IP) injection for three days after irradiation. Three days after irradiation, all rats were sacrificed and their livers were excised to measure the biochemical parameters malondialdehyde (MDA) and glutathione (GSH). Each data point represents mean ± standard error on the mean (SEM) of at least eight animals per group. A one-way analysis of variance (ANOVA) was performed to compare different groups, followed by Tukey’s multiple comparison tests (p<0.05).

**Results:**

The results demonstrated that whole body irradiation induced liver tissue damage by increasing MDA levels and decreasing GSH levels. Hepatic MDA levels in irradiated rats that were treated with melatonin (30 mg/kg) were significantly decreased, while GSH levels were significantly increased, when compared to either of the control groups or the melatonin only group.

**Conclusion:**

The data suggest that administration of melatonin before and after irradiation may reduce liver damage caused by gamma irradiation.

## Introduction

Ionizing radiation interacts with biological systems to induce excessive fluxes of free radicals that attack various cellular components including the DNA, proteins, and membrane lipids, leading to significant cellular damage (1, 2). Radiation-induced liver disease (RILD) is a dose-limiting complication in treatment. Therefore, the treatment options for RILD are limited, and in severe doses of irradiation, liver failure and death can occur (3-5).

Lipid peroxidation is believed to be an important cause of destruction and damage to cell membranes and has been suggested to be a contributing factor to the development of oxygen radical-mediated tissue damage (6). Radiation-induced lipid peroxidation is a free radical process (7). Since these radicals initiate lipid peroxidation, it is expected that patients who undergo whole body radiation will have high levels of lipid peroxidation. Malondialdehyde (MDA) and lipid peroxides are products of the lipid peroxidation process. MDA with high cytotoxicity and inhibitory actions on protective enzymes, acts as a tumor promoter and a co-carcinogenic agent (6). Koc et al. (6) showed that total body irradiation (TBI) of rats resulted in a significant increase in liver tissue MDA levels and decrease of antioxidant enzymes activities.

The antioxidant system consists of low-molecular- weight antioxidant molecules, such as glutathione (GSH) and various antioxidant enzymes (8). Glutathione is one of the most important molecules in the cellular defense against chemically reactive toxic compounds that induce oxidative stress, while depletion of tissue GSH is one of the primary factors that permits lipid peroxidation to occur. Decreased cellular GSH levels and reduced capacity for GSH synthesis sensitize cells to radiation injury (8). Sener et al. (9) showed that whole body irradiation of rats resulted in a significant decrease in the liver tissue GSH levels.

Melatonin (N-acetyl-5-methoxytryptamine) is an endogenous compound synthesized by the pineal gland in the human brain (10-12). Studies have shown the different features that make melatonin a potentially useful radioprotector, such as the direct scavenging of free radicals; the ability to stimulate the activity of antioxidant enzymes and to inhibit the activity of a pro-oxidative enzyme; distribution in all tissues, cells,and cellular compartments throughout the organism; quick diffusion through all biological membranes; ability to provide radioprotection without receptor interaction; tolerance to high doses of melatonin by living organisms and little toxicity in the species tested; ease of oral administration; etc. (13).

These mechanisms require the presence of melatonin at the time the cells and tissues are irradiated (13, 14). The widespread distribution of melatonin in subcellular compartments such as cytosol, nucleus, mitochondria, and cellular membrane have allowed it to effectively protect various normal cells from oxidative damage induced by ionizing radiation (12, 15).

Vijayalaxmi et al. (16-18) reported that melatonin protects or reduces gamma radiation-induced chromosome damage, micronuclei and primary DNA damage in human peripheral blood lymphocytes in their *in vitro* and *in vivo*/ *in vitro* investigations. Melatonin also increases intracellular glutathione (GSH) levels by stimulating the synthesis of the rate-limiting enzyme, γ-glutamylcysteine synthase, which inhibits the pero-oxidative enzymes nitric oxide synthase and lipoxygenase (13). There is also some evidence that melatonin stabilizes microsomal membranes and thereby probably helps them resist oxidative damage (19). Moreover, the radio-protective effect of melatonin against organ damage induced by irradiation has been reported by Sener et al. (9) and El-Missiry et al. (20).

In this study the antioxidant role of melatonin against oxidative damage caused by gamma irradiation in liver tissue after whole body radiation was investigated.

## Materials and Methods

### Chemicals

All reagents were of the highest quality available. Melatonin was obtained from sigma Chemical Co. (St. Louis, MO, USA) and other chemicals used in this study were obtained from both sigma (St. Louis, MO, USA) and Merck (Germany).

### Animals

Adult male albino wistar rats weighing 200- 250 g were obtained from the Experimental Animal Laboratory section of the Department of Pharmacology, Tehran University of Medical Sciences, and were housed in stainless steel cages and supplied with wood chips, in a temperature controlled room (22℃) and a 12 hour light-dark cycle. The animals were allowed free access to tap water and a standard diet for the duration of the study. The experimental protocol was in accordance with the guidelines for care and use of laboratory animals as adopted by the Ethics Committee of the School of Medicine, Tehran University of Medical Sciences, Tehran, Iran.

### Experimental design

In this experimental study, thirty-two rats were divided into four groups. Group 1 did not receive melatonin or irradiation (Control group) but received 500 µL isotonic NaCl solution, as a vehicle, intraperitoneally (IP), group 2 (Mel group) only received melatonin (30 mg/kg on the first day and 30 mg/kg melatonin daily through intraperitoneal injection for three days after irradiation), group 3 received 500 µL isotonic NaCl solution (IP) and was exposed to 10 Gy whole body gamma irradiation (10 Gy group), and group 4 received 30 mg/kg melatonin (IP) 30 minutes prior to radiation, plus whole body irradiation of 10 Gy plus 30 mg/kg melatonin daily through IP injection for three days after irradiation (Mel + 10 Gy group).

Rats in groups 2 and 4 were given an IP injection of freshly prepared melatonin in 500 µL of 10% absolute ethanol solution. Melatonin was first dissolved in a small amount of absolute ethanol (50 µL) and then diluted with isotonic NaCl solution to givea final ethanol concentration of 10%.

Animals were followed for 3 days after radiation during which melatonin (30 mg/kg) injections were repeated, for groups 2 and 4, once daily. At the end of this period, all rats were sacrificed and their livers were excised to measure the biochemical parameters i.e. MDA and GSH.

### Irradiation

All of the rats were anesthetized with an IP injection of ketamine (50 mg/kg) and chlorpromazine (10 mg/kg), and then the rats in groups 3 and 4 were exposed to a whole-body gamma radiation dose of 10 Gy. Irradiation was performed using a cobalt-60 teletherapy unit (Theratron 780, Atomic energy of Canada limited, Canada) at a dose rate of 50cGy/minute using the Single-wavelength anomalous dispersion (SAD) method [SAD: 80 cm, Field (at SAD=80 cm): 35 cm 35 cm].

### Tissue preparation and biochemical analysis

Three days after irradiation the rats were anesthetized and the abdomen opened immediately for access to and examination of the liver. After opening the abdomen, 1ml of 0.9% cold saline was injected into the portal vein. The liver was excised and blot dried. 400 mg of the left lower lobe of the livers was homogenized in 1 ml of 0.9% cold saline. Then, 0.2 ml of 25% trichloroacetic acid (TCA) was added to the homogenate and centrifuged at 5000 rpm for 15 minutes. The clear upper supernatant was used for measuring GSH content and the sediment was used for measuring the MDA level.

### MDAanalysis

The MDA level was determined according to the thiobarbituric acid (TBA) method (21). Briefly, 2.5 ml of 0.05 M sulfuric acid and 3 ml of a 0.2% solution of TBA were added to the sediment. The mixture was heated at 100℃ for 30 minutes in a boiling water bath. Four cubic centimetres of n-butanol were added to the cooled mixture and the sample was shaken vigorously. After centrifugation at 3500 rpm for ten minutes, the organic layer was removed and its absorbance read at 532 nm. The MDA concentration was calculated from the standard curve. Tetraethoxypropane (TEP) was used as the standard for setting up the calibration curve.

### GSH analysis

GSH level was determined according to the method of Kuo and Hook (21). Briefly, 0.5 cc distilled water, 2 cc of 0.3 M disodium phosphate (Na_2_HPO_4_) and 0.5 cc of 0.04% 5, 5'-dithiobis- 2-nitrobenzoic acid (DTNB) were added to 0.5 cc of the supernatant and incubated for ten minutes at room temperature. The absorbance of the resulting yellow color was read against the blank at 412 nm and the GSH concentration was calculated from the standard curve. Pure GSH was used as the standard for establishing the calibration curve.

Biochemical measurements were carried out at room temperature using a spectrophotometer (Pharmacia Biotech, Cambridge, UK).

### Statistical analysis

Each data point represents the mean ± standard error of the mean (SEM) of at least eight animals per group. A one-way analysis of variance (ANOVA) was performed to compare different groups, followed by Tukey’s multiple comparison tests. P < 0.05 was considered to represent a statistically significant difference.

## Results

### MDA levels

**Fig 1:**
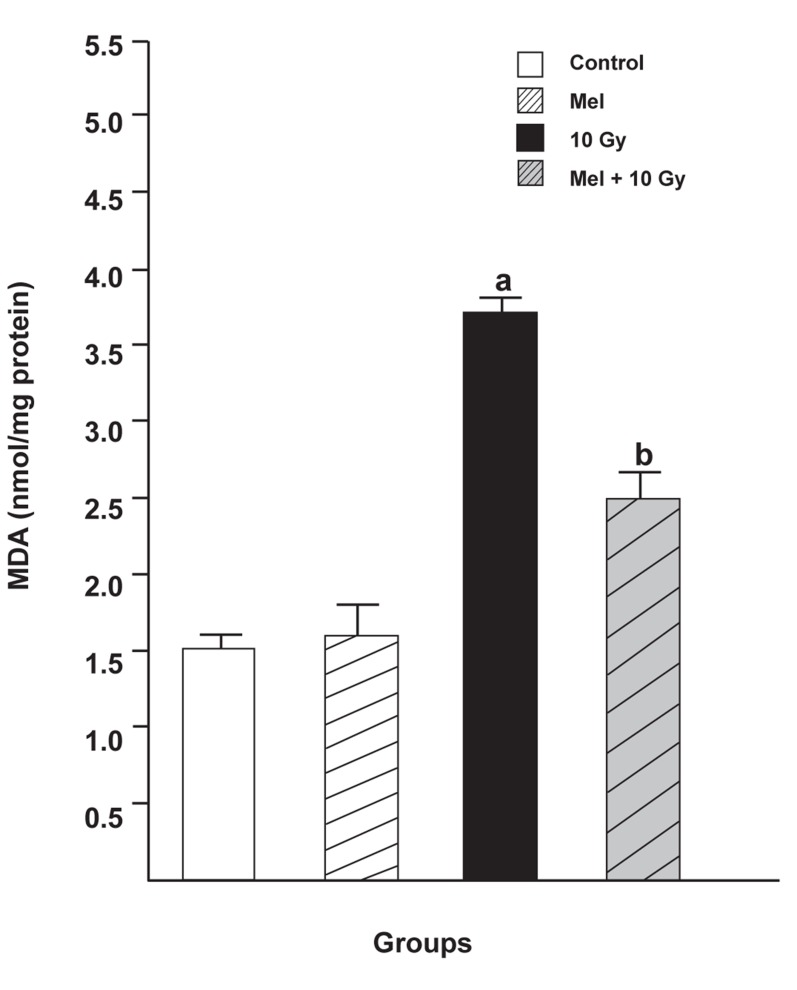
The effect of melatonin on MDA levels in rats subjected to whole body gamma irradiation. Data represent mean ± standard error ofthe mean (SEM) (n=8 animals per group). a; P<0.05 (compared to control group), and b; P<0.05 (compared to the radiated groups).

As can be seen in figure 1, MDA levels in the irradiated only group (3.7180 ± 0.1076, p <0.05) were significantly higher compared with either the control group (1.5080 ± 0.2676, p <0.05) or the melatonin only group (1.6000 ± 0.2267, p <0.05). Melatonin pretreatment and treatment significantly reduced MDA levels in the livers of rats subjected to whole body irradiation (2.5040 ± 0.1698, p <0.05).

### GSH levels

**Fig 2:**
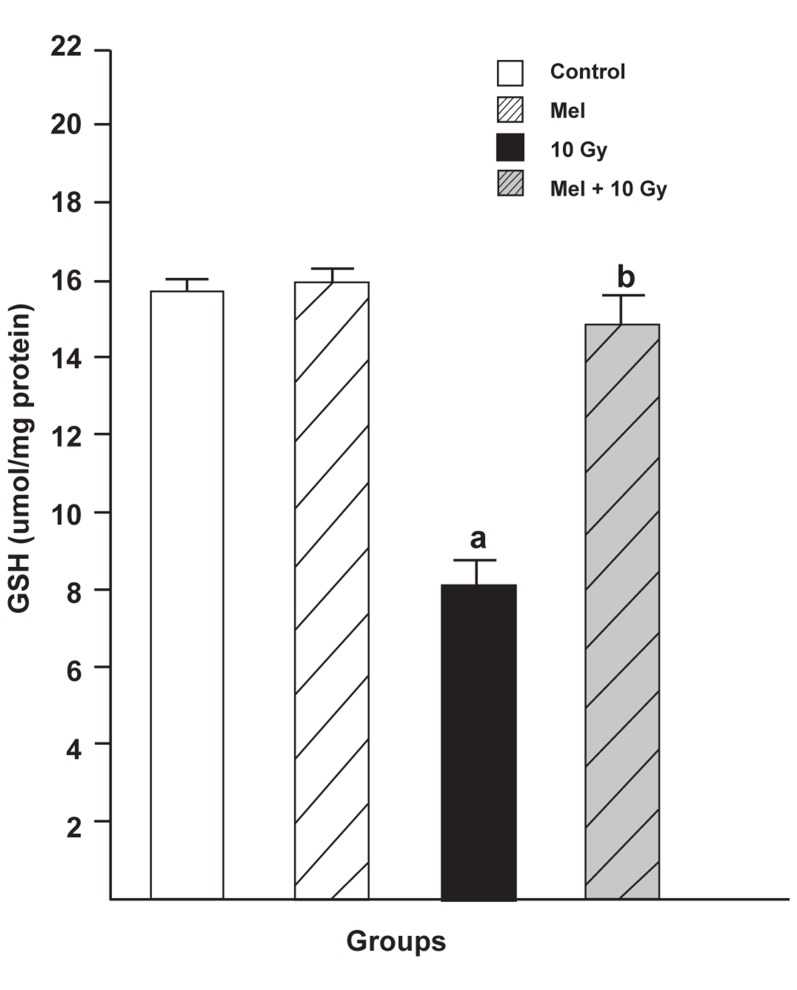
The effect of melatonin on GSH levels in rats subjected to whole body gamma irradiation. Data represent mean ± standard error of the mean (SEM) (n=8 animals per group).a; P<0.05 (compared to control group), and b; P<0.05 (compared to the radiated groups).

As shown in figure 2, the levels of GSH in the liver tissues significantly decreased in the irradiated only group (8.194 ± 0.717, p <0.05) when compared to either the control group (15.836 ± 0.316, p <0.05) or the melatonin only group (16.060 ± 0.427, p <0.05). Melatonin pretreatment and treatment significantly reversed the GSH levels of rats exposed to whole body irradiation (14.946 ± 0.841, p < 0.05).

## Discussion

Several studies have demonstrated that melatonin appears to ameliorate irradiation-induced injury in various organs including the spleen (8, 11, 22), liver (5, 9, 20), lung, colon, ileum (9) , lens (23, 24), spinal cord (15, 25), and brain (26). Melatonin synergistically acts as an immunostimulator (22, 27) and antioxidant (10, 14, 28, 29). Moreover, due to small size and high lipophilicity, melatonin crosses biological membranes and reaches all compartments of the cell (28). Melatonin has been shown to be a direct free radical scavenger and indirect antioxidant via its stimulatory actions on antioxidant enzyme activity (10, 12, 27, 29, 30) and inhibitory actions on pro-oxidative enzyme activity(13, 27, 31).

The results of the present study suggest that whole body irradiation caused tissue damage in the rat liver as manifest by increased MDA levels and decreased GSH levels. The increase in MDA levels in the irradiated group demonstrates the role of oxidative mechanisms in the irradiation induced tissue damage. Due to high cytotoxicity and inhibitory actions on protective enzymes MDA acts as a tumor promoter and a cocarcinogenic agent (6).

As mentioned earlier, GSH provides considerable protection against oxidative injury through its role in the cellular system of defense against oxidative damage. The decrease in tissue GSH levels after whole body irradiation may be due to its consumption during the oxidative stress induced by irradiation. On the other hand, melatonin administration before and after radiation, alleviated the radiation toxicity to the liver by reversing the radiation-induced effects. The mechanisms whereby melatonin inhibits lipid peroxidation probably include the direct scavenging of the initiating radicals, especially •OH and ONOO¯(7).Also, melatonin through its indirect antioxidative properties stimulates levels of GSH which is an antioxidant.

Several studies have indicated that tissue injuries, induced by various stimuli, are coupled with GSH depletion and high levels of lipid peroxidation (32, 33). Also,research in the last decade has demonstrated that melatonin, by its free radical scavenging and antioxidative properties, ameliorates radiation toxicity in different tissues. Koc and colleagues investigated the antioxidant role of melatonin (at 5 and 10 mg/ kg) against whole body gamma irradiation induced oxidative damage in liver tissue with a single dose of 6Gy (6). The results demonstrated that in irradiated rats, pretreated with melatonin (5 or 10 mg/ kg), liver tissue MDA levels, as an end product of lipid peroxidation, were significantly lowered, whereas the activities of superoxide dismutase (SOD) and glutathione peroxidase (GSH-Px), two of the most important antioxidant enzymes, were significantly increased. The authors concluded that pretreatment with melatonin may prevent irradiation induced liver damage (6). El-Missiry et al. (20) showed that treatment with 10 mg/kg melatonin for 4 days (daily) before acute irradiation (2 and 4Gy) significantly reduced radiationinduced elevations in MDA and protein carbonyl levels(the oxidative stress markers) in the liver and maintained hepatic glutathione (GSH) content, glutathione-S-transferase (GST), and catalase (CAT) activities close to the control group values.

Sener et al. (9) evaluated the levels of MDA and GSH in liver, lung, colon and intestinal tissues. The results demonstrate that both 12 hours and 72 hours following irradiation, tissue levels of MDA were elevated, while GSH levels were reduced in liver and other organs. On the other hand, melatonin reduced the levels of MDA and increased the GSH levels significantly (9).

radio-protective effects of melatonin on biochemical, histopathological, and clinical manifestations of radiation myelopathy (RM) in the rat cervical spinal cord. Administration of melatonin markedly reduced MDA and increased GSH levels when compared with the control group (15). Similarly, new data from another study have shown that radiation exposure decreased levels of GSH and increased levels of MDA in the lens, but these values were within normal limits when melatonin was administered (23).

In our current study, results for the irradiation plus melatonin groups were similar to the results of previous studies in which treatment with melatonin increased GSH levels but decreased levels of MDA. Thus, our results are in agreement with other studies and support findings previously published in the literature (6, 9, 15, 20, 22).In our most recent study prior to this one, we investigated the possible radio-protective effects of melatonin (10 mg/kg) against whole body irradiation (2 and 8 Gy) induced oxidative damage to rats peripheral blood at different time points after exposure. Treatment with melatonin (10 mg/kg) ameliorated the harmful effects irradiation by increasing the lymphocyte count (LC) and antioxidant enzyme activity, and decreasing nitric oxide (NO) levels at all time points (27). We concluded that 10 mg/kg melatonin is likely to be an adequate concentration for significant protection against the lower dose of 2 Gy but does not provide significant protection against the higher dose of 8 Gy. It seems, therefore, that the radio-protective effects of melatonin are dose-dependent (27).

Despite the lack of clinical and experimental studies, other investigators findings taken together with present results and our previous studies (15, 23, 25, 27, 34) suggest that administration of melatonin may enable the use of higher doses of irradiation during radiotherapy and may be beneficial in alleviating the complications of cancer treatment.

## Conclusion

Based on our results, ionizing radiation causes oxidative damage while melatonin, due to its antioxidative properties, ameliorates radiation induced injury to the rat liver. In conclusion, our results show that a concentration of 30 mg/kg melatonin is likely to provide significant protection against 10 Gy gamma irradiation of rat liver.These findings hold the promise that in future higher concentrations or long-term administration of melatonin may provide more protection against higher doses of irradiation induced oxidative stress in various organs. Further experiments and clinical trials remain necessary to validate these findings.
